# Questionable and Improved Research Practices in Single-Case Experimental Design: Initial Investigation and Findings

**DOI:** 10.1007/s40614-025-00441-9

**Published:** 2025-03-14

**Authors:** Matt Tincani, Jason Travers, Art Dowdy, Timothy A. Slocum, Ronnie Deitrich

**Affiliations:** 1 Department of Teaching and Learning, Temple University, Philadelphia, PA USA; 2 Department of Special Education and Rehabilitation Counseling, Utah State University, Logan, UT USA; 3 The Wing Institute at Morningside Academy, Seattle, WA USA

**Keywords:** Single-case experimental design, Questionable research practices, Improved research practices, Open science practices, Quality research indicators

## Abstract

Researchers have identified questionable research practices that compromise replicability and validity of conclusions. However, this concept of questionable research practices has not been widely applied to single-case experimental designs (SCED). Moreover, to date researchers have focused little attention on improved research practices as alternatives to questionable practices. This article describes initial steps toward identifying questionable and improved research practices in SCED. Participants were 63 SCED researcher experts with varying backgrounds and expertise. They attended a 1-day virtual microconference with focus groups to solicit examples of questionable and improved research practices at different stages of the research process. A qualitative analysis of over 2,000 notes from the participants yielded shared perspectives, resulting in 64 pairs of questionable and improved research practices in SCED. Our results highlight the need for further evaluation and efforts to disseminate improved research practices as alternatives to questionable practices.

Science is a process in which understanding of the natural world improves over time through systematic observation and, when possible, controlled manipulation (Sidman, 1960; Skinner, 1956). Scientific knowledge increases as previous understanding is refined or replaced with more powerful and precise understanding. This process requires ongoing critical appraisal of current understanding and openness to newer formulations (Popper, 2005). Improved scientific understanding is driven, in part, by parallel advancements in conducting, reporting, and vetting research (Nosek et al., 2022). Refining research methods requires critique and evaluation of current methods in the service of further improvement (e.g., O’Donohue et al., 2016). Recognition of methodological limitations in current practice and improvement in methods is not a weakness, but rather a core strength of science.

Scientific methods are perpetually in flux as advances are discovered and adopted, but over the past two decades there has been increased concern about scientific fields that rely on group comparisons research methods. In particular, researchers across disciplines have sought to replicate previously published studies and found the findings less reliable or robust than initially conceived, or they have noted a general lack of published replication studies (e.g., Evanschitzky & Armstrong, 2010; Hubbard & Armstrong, 1994; Ioannidis, 2005, 2012; Open Science Collaboration, 2015; Makel & Plucker, 2014; Pashler & Harris, 2012). In one highly publicized example, the Open Science Collaboration (2015) sought to replicate findings of 100 studies published in three well-known psychology journals. On average, the replication attempts yielded only half the mean effect sizes of the original studies, and expert raters judged only 39% of replication study effects as consistent with original study results. Overall, the “replication crisis” has been identified as one of the foremost challenges to scientific integrity and credibility of psychological science within the public sphere (Anvari & Lakens, 2018; Nosek et al., 2022).

## Questionable Research Practices

Questionable research practices (QRPs) have been identified as a key contributor to a replication crisis in numerous scientific fields, including psychology and education (Anvari & Lakens, 2018; O’Donohue & Masuda, 2022; Pashler & Wagenmakers, 2012; Simmons et al., 2011). There is no ubiquitous definition nor a comprehensive list of QRPs; however, John et al. (2012) and others (e.g., Makel et al., 2021) have compiled representative lists. Table 1 contains a list and description of common QRPs.

**Table 1:** Examples of Commonly Identified Questionable Research Practices (QRPs)

Questionable Research Practice	Description
Selective Data Reporting	Selectively reporting positive/statistically significant results while omitting negative/insignificant results (Franco et al., 2014; Rosenthal, 1978)
*p*-hacking	Selectively conducting data analyses to produce and/or enhance positive/statistically significant outcomes (Head et al., 2015)
HARKing (Hypothesizing After the Results are Known)	Formulating hypotheses after study outcomes are obtained to “fit” the data (Kerr, 1988)
Selective Procedural Reporting	Omitting possible confounds from procedural descriptions that could explain positive/statistically significant outcomes (Ludwig et al., 2023)
Selective Outcome Reporting	Writing a paper’s abstract or discussion to selectively downplay undesirable results and/or emphasize desired results (Gerrits et al., 2019)
Selective Recruiting	Selectively recruiting participants into a treatment condition who are more likely to show positive effects of the treatment and not reporting this (O’Donohue et al., 2016)

Examination of the QRPs in Table 1 highlights that some QRPs can occur regardless of the type of design used. For instance, selective data reporting through omitting some or all of a participant’s data can happen in either group or SCED studies (e.g., Tincani & Travers, 2022). However, discussion about causes of the replication crisis and its relationship to QRPs has primarily been focused on group comparisons research with null-hypothesis statistical significance testing (e.g., Reis & Friese, 2022). The behavioral processes that shape and maintain QRPs likely affect all scientists (Clark et al., 2022); thus it seems likely that QRPs also occur in SCED. However, SCED entails substantially different research methods and data analysis strategies so the degree to which QRPs for group comparisons research apply to SCED is not clear.

**Fig. 1:**
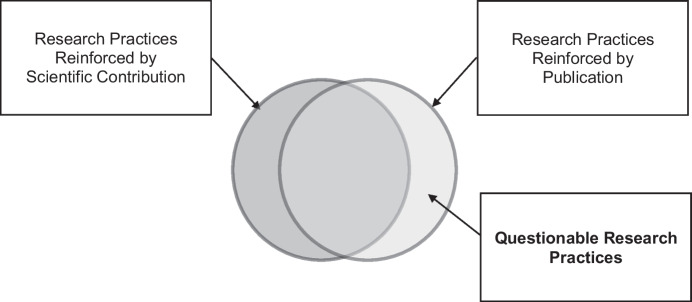
Contingencies of Questionable Research Practices

Group comparisons researchers test a priori hypotheses with inferential statistical methods that can be manipulated to attain expected or preferred outcomes (Makel et al., 2021). For example, *p*-hacking is comprised of various strategies to create or enhance the appearance of statistically significant effects, such as by running several different analyses and selectively reporting those that yield significant *p* values (Head et al., 2015). In contrast, SCED researchers predominantly rely on visual analysis to interpret data and therefore may be unlikely to engage in QRPs like *p*-hacking. Instead, an SCED researcher may engage in different practices that result in the appearance of robust visual effects. These may include altering the scale of a graph’s x- and y-axes, omitting datapoints from graphs indicating instability, selectively parsing or combining dependent variables in graphs, or selectively depicting data in bar graphs that emphasize central tendency relative to line graphs that provide a more nuanced depiction of behavioral variability (Tincani & Travers, 2022). Despite differences in the topography of QRPs, group comparisons researchers have identified variables that support a functional analysis of researcher behavior (e.g., Nosek et al., 2012; Simmons et al., 2011), and it seems likely the contingencies that motivate QRPs for group researchers are also relevant to SCED researchers (see Fig. 1).

Researcher behavior is shaped by multiple overlapping contingencies as highlighted by the conceptual diagram in Fig. 1. One set of maintaining consequences, denoted by the circle on the left, involves making contributions to a robust and valid research literature that extends scientific understanding and practical utility. As denoted by the circle on the right, researcher behavior is also shaped by consequences associated with volume of publication and associated metrics, which often confers promotion, prestige, and other tangible benefits, such as grant funding (Lilienfeld, 2017; Marsicano et al., 2022; Schimanski & Alperin, 2018). Such contingencies can operate on researchers and influence their decisions before, during, and after a study is conducted. The relative contribution of each set of contingencies varies according to a host of researcher- and study-specific variables; however, when researcher behavior is controlled primarily by contingencies related to publication, as denoted by the nonoverlapping area in the circle on the right, QRPs can result.

Simmons et al. (2011) highlighted how researchers often have many decisions to make, or degrees of freedom, when conducting a study. They identified several degrees of freedom including whether more data should be collected, some observations should be excluded, conditions should be combined or compared, control variables should be considered, and specific measures should be combined, transformed, or both (see p. 1359). They noted researcher decisions cannot always be anticipated prior to a study and therefore cannot be planned in advance, nor are there clear-cut rules for how best to make such decisions in most research situations. However, when these degrees of freedom enable researcher behavior primarily reinforced by publication, but less so by scientific contribution, QRPs may occur.

For example, group comparisons researchers may differentially select analytic approaches that are more consistent with their pre-study hypotheses or analyze data in ways that yield clear and unambiguous findings more likely to be published. Likewise, SCED researchers may graphically depict their results, combine dependent variables, or scale graph axes to present data that support a priori hypotheses or increase probability their research will be published (Tincani & Travers, 2022; Tincani et al., 2024). This analogy suggests that group comparison and SCED researchers are subject to similar contingencies, and that for both groups researcher degrees of freedom may serve as context for behavior (i.e., QRPs) that produces less valid research reports. For clinicians who rely on applied research for evidence-based treatments, QRPs can result in wasted resources and, at worst, iatrogenic (i.e., countertherapeutic) effects. 

### Questionable Research Practices in SCED

This conceptual analysis of contingencies does not constitute evidence that SCED researchers engage in QRPs. There exists, however, some evidence of QRPs in SCED research. One form of evidence is based on researcher evaluations of datasets that demonstrate varying degrees of experimental control. Shadish et al. (2016) provided single-case research experts with hypothetical datasets that demonstrated varying degrees of experimental effect and asked them to complete a questionnaire about their interpretations. A majority of researchers reported they were more likely to recommend publication of a submitted manuscript when a dataset showed positive effects, and some researchers indicated a willingness to exclude datasets showing weaker experimental effect from their manuscripts submitted for publication. These findings suggest that research is more likely to be published when effects are large and consistently positive, and less likely to be published when effects are mixed or less pronounced.

A second form of evidence comes from systematic reviews comparing results of intervention studies described in published journal articles to those of unpublished reports, such as doctoral dissertations, that are searchable through databases or otherwise discoverable (see Polanin et al., 2016). If the body of published studies yield larger effects than the set of unpublished ones, this arguably suggests evidence of selective data reporting (i.e., a QRP related to the file drawer effect; Pigott et al., 2013; Rosenthal, 1978; See Table 1). For example, Sham and Smith (2014) compared effect sizes for published and unpublished SCED studies on a behaviorally based intervention called pivotal response treatment (Koegel & Koegel, 2006). Sham and Smith found published studies of pivotal response treatment had larger treatment effects than unpublished studies. Likewise, Dowdy et al. (2020) reported larger effect sizes for the set of published studies of response interruption and redirection when compared to published journal articles. This finding suggests that there may be a systematic tendency to selectively publish results that show larger effect sizes and that this may bias the body of SCED research on socially important interventions.

A third way to evaluate selective reporting is to compare data reported from dissertations to data reported in corresponding journal articles based on those dissertations. Selective reporting is found when authors omit results, including those reflecting weaker experimental effect, from the published report. For example, Travers et al. (under review) investigated selective reporting in SCED research by comparing results from dissertations that used SCEDs to the corresponding published article (the project’s OSF page can be found here). Of the 124 dissertation-article pairs compared, 12.4% of articles omitted one or more participants and/or dependent variables from the corresponding dissertation. They also found published studies had a higher proportion of experimental effects to noneffects and larger effect sizes when compared to dissertations. The authors concluded that selective reporting appears to occur in SCED studies, and that omission of results may inflate perceived effects in ways that mislead researchers and professionals.

Overall, these findings suggest that the SCED literature is likely affected by QRPs that involve selective reporting. Although there is insufficient evidence to declare a “replication crisis,” as has been reported in other disciplines (e.g., Nosek et al., 2022), these data highlight salient concerns about the potential presence of QRPs in SCED research and the need for additional investigation. In the next section, we describe efforts to address QRPs through open science practices and the use of quality research indicators, and how these are likely insufficient to prevent all instances of QRPs in SCED.

### Open Science Practices and Quality Indicators to Prevent Questionable Research Practices

Given that QRPs occur in various scientific domains, researchers across disciplines have proposed alternative practices to prevent them, most prominently those based in the open science movement (Cook et al., 2018; Johnson & Cook, 2019; Nosek et al., 2012; Nosek et al., 2018). For example, preregistration, an open science practice that involves publicly documenting study procedures and planned analyses prior to conducting a study, has been proposed as a means for preventing some types of QRPs (e.g., *p*-hacking and other post-hoc data manipulation; Simmons et al., 2021). In particular, when procedures and analysis plans are published prior to conducting a study, researchers have fewer degrees of freedom to manipulate statistical tests after data have been collected. Other open science practices emphasize transparency during all stages of the research process and include making readily available all data and materials, open (i.e., unblinded) peer review, open access publication, posting pre-prints, team science, and others (Munafò et al., 2017). Such transparency could conceivably prevent some QRPs in SCED (Tincani et al., 2024).

The promise of open science practices for preventing specific QRPs notwithstanding, there are important caveats to consider. Although advocates have made a conceptual case for open science practices (e.g., Cook et al., 2018; Tincani et al., 2024), empirical evidence supporting the benefits remains limited. Some investigators have reported open science practices have positive effects on methodological quality and rigor of research (e.g., Soderberg et al., 2021), but others have found equivocal effects of open science practices on QRPs (e.g., van den Akker, 2023). This discrepancy implies that some open science practices may not sufficiently discourage or prevent QRPs. Moreover, certain open science practices are intended to increase public access to research such as through preprints and open access journals, but these practices are not targeted at reducing QRPs. Thus, it appears additional strategies beyond open science practices are needed to fully address QRPs.

Likewise, researchers have proposed various quality standards for SCED research that address methodological rigor and replicability. These include the completeness of descriptions of participants and settings, sufficiency of experimental designs for demonstrating experimental control, and the validity of measurement and observation systems (Cook et al., 2014; Horner et al., 2005; Kratochwill et al., 2023). When researchers use methodologically rigorous SCEDs, they also may avoid engaging in certain QRPs. For example, researchers who completely describe features of the context or setting where a study occurred also disclose potential confounds that inflate the appearance of therapeutic effects, including aspects of an educational environment, apart from the intervention, that facilitate learning (see Table 1). However, quality indicators and other standards may not be sufficiently specific or may not address some types of QRPs. For example, standards and quality indicators for SCED have not addressed issues of selective reporting or clearly distinguishing a priori decisions from those made during or after data collection, which are potentially important types of QRPs. Thus, methodological quality indicators and open science practices may only be partially effective strategies for preventing QRPs. However, this is a tentative hypothesis with limited direct evidence; it highlights the need to systematically identify practices that are considered questionable, along with corresponding alternative practices, by the SCED research community.

### Improved Research Practices

We have argued that QRPs are methodological decisions motivated and reinforced by contingencies of publication that can distort conclusions drawn from the scientific literature. We also described some evidence that QRPs may occur in SCED research, and that certain QRP topographies likely are unique to SCED research (see also Tincani & Travers, 2022). Given limited research on QRPs in SCED, there is a need for additional empirical data identifying QRPs that are relevant to SCED.

However, problem identification is only the first step in generating behavior change. To be useful, an analysis of behavior must result in interventions that support desirable responses (Carr, 1977). Thus, it is important to also identify more desirable, improved research practices (IRPs) to replace QRPs and promote a more robust and valid research base. For example, if selectively reporting participants and/or variables is considered to be a QRP by the SCED research community, then one potential IRP might be briefly reporting the number of excluded participants and variables, along with the rationale, and making available the full dataset in an online supplement. Likewise, if SCED researchers believe it problematic to selectively recruit participants most likely to respond to an intervention without disclosing this activity, then a potential IRP might be to explicitly report the known participant characteristics that likely affected responsiveness to intervention (e.g., relevant prerequisite skills, history with related interventions).

### Purpose of the Project

QRPs are widely considered primary contributors to the replication crisis in disciplines that rely on group comparisons designs (e.g., Makel et al., 2021). SCED is a substantially different research method that may be subject to a distinct set QRPs. Open science practices and methodological quality indicators appear to be complementary but insufficient strategies for limiting QRPs in SCED. If so, researchers may benefit from a systematically derived list of QRPs and IRPs for SCED research. Such a list could help reduce bias, further strengthen the validity of SCED results, and enhance the perceived value of SCED findings by consumers and the broader scientific community. The overall purpose of the research reported here was to identify QRPs and corresponding IRPs in SCED through attaining diverse input from the SCED research community.

## Method

**Fig. 2:**
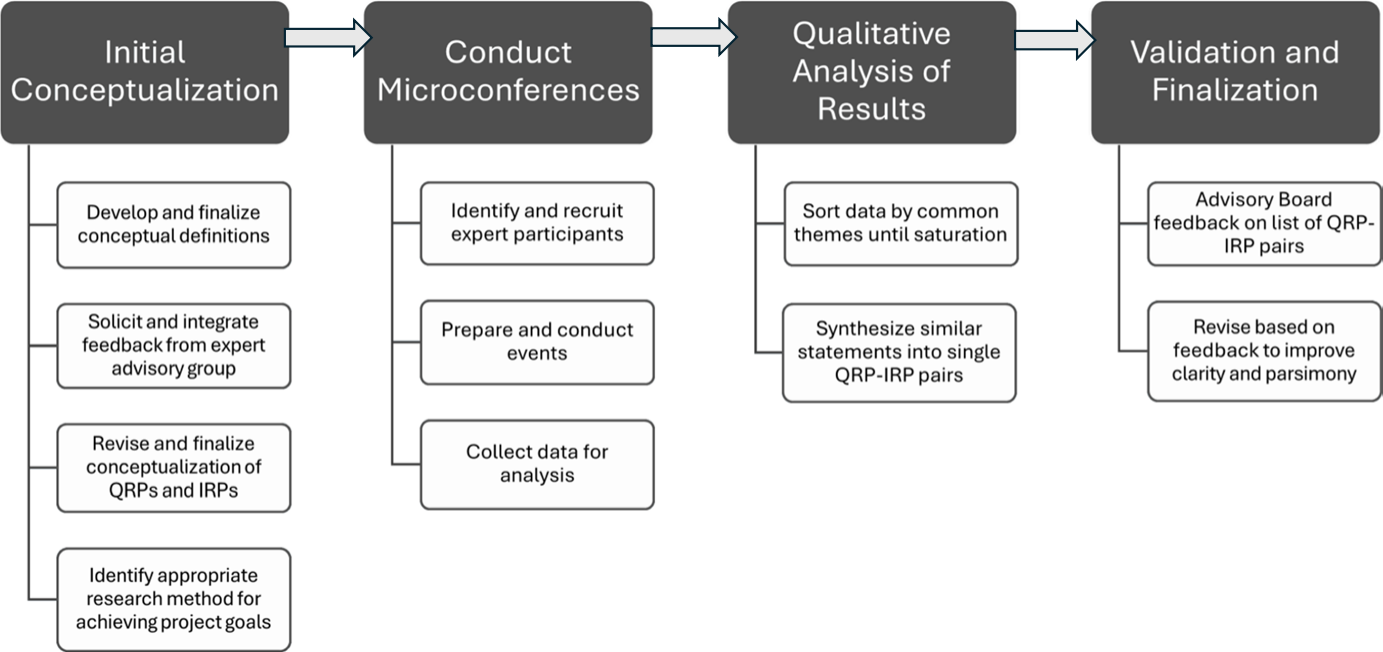
Approach to Develop a Preliminary List of Questionable Research Practices and Improved Research Practices. *Note*. QRP = Questionable Research Practice; IRP = Improved Research Practice

Our approach was comprised of the steps illustrated in Fig. 2. First, we solicited advice and opinions from a small advisory group of widely recognized senior experts on SCED on our conceptual framework and procedures for our approach. With this input, we conducted a series of online microconferences to solicit input from a large group of SCED experts representative of various research and practice domains. We then conducted a qualitative analysis of their responses to develop a preliminary list of QRP-IRP pairs representing their statements about QRPs and IRPs. Following an iterative refining process conducted by the first four authors, results of our analysis were presented to our advisory board in the form of a written summary provided in advance of a virtual meeting and, with their additional feedback, was revised and finalized.

### Participants

The purpose of online microconferences was to generate a preliminary yet comprehensive list of QRPs and associated IRPs for SCED research. Thus, we recognized a need to include a broad group of SCED researchers representing the range of contexts addressed by SCED to increase the probability of generating a comprehensive list of research practices. Following approval from a university institutional review board, we recruited participants who represented the various scientific fields where SCED is often published. We generated a list of researchers to invite based on several key considerations including academic discipline (e.g., applied behavior analysis, special education, school psychology, speech-language pathology), career stage (i.e., early, mid-, and advanced career stage), roles and responsibilities (e.g., editors, textbook authors, methodologists), and demographic characteristics (e.g., gender, race, age). We sent email invitations to 115 researchers with expertise in SCED. The invitation included a two-page overview of the purpose, goals, and procedures for the microconferences. A total 63 (55%) individuals accepted our invitation to participate one of the four microconferences (a list of microconference participants who wished to be acknowledged for their participation can be found on the project’s OSF site: https://osf.io/mvy6c). Participants’ self-identified disciplinary affiliations were as follows (they could select more than one): applied behavior analysis (*n* = 31), special education (*n* = 27), school psychology (*n* = 15), experimental analysis of behavior (*n* = 5), other (e.g., research methodology and statistics; *n* = 5), and speech-language pathology (*n* = 1). Women accounted for 27 (43%) of the participants and 4 (6%) identified as a person of color. Given the scope of their participation (i.e., one 6-h microconference and preconference preparation of approximately 2-h in length), we compensated each attendee $500 for their participation.

### Microconference Format and Process

Each microconference was a 6-h (1-day) virtual meeting of 16–18 SCED experts with the goal of generating a list of QRP-IRP pairs. The microconferences were conducted in the summer of 2022 on July 19 (*n* = 16), July 20 (*n* = 16), July 21 (*n* = 16), and August 18 (*n* = 18). Approximately 4 weeks prior to each microconference, we sent each participant a worksheet with instructions to prepare for the event. The worksheet contained a list of QRPs commonly identified in the group comparisons literature (e.g., Makel et al., 2021) with instructions for participants to identify their potential relevance to SCED and possible analogous practices in SCED, along with possible IRPs corresponding to each QRP. Participants were also provided with two readings (Nosek et al., 2012; Tincani & Travers, 2022) on topics related to QRPs to help contextualize their participation. These materials are available on the project’s open science project page (https://osf.io/mvy6c). Finally, participants received a link to a brief video that explained how to use Google Jamboard, the online platform we used to facilitate brainstorming and record participant responses.

Each microconference was held on the videoconferencing platform Zoom and began with introductions of the authors and the advisory board members, each of whom were experts in SCED (10 min). Next, we spent approximately 20-min describing foundational information about QRPs and related concepts followed by the purpose of our project and question/answer from the participants. We then used 30 min to overview related conceptual frameworks (e.g., risk of bias, quality indicators) and lead a guided discussion with participants. This was followed by 10-min explanation of the primary activity of the conference: Generating lists of QRPs and IRPs that may occur during each phase of a research study (e.g., recruiting, selecting, assigning and reporting participants; see below). Participants were then divided into breakout groups of three to five to identify, discuss, and clarify QRPs. Each group was facilitated by one of the first four authors. The breakout groups lasted a total of 3-h 20-min, with a 30-min break at the halfway point.

During each session, the facilitator followed a script to guide participant brainstorming. First, they briefly overviewed the procedures for the session and shared a link to a Google Jamboard file. Jamboard is a web-based app similar to a bulletin board that enables users to simultaneously post digital “sticky notes” that all group members could see. We chose this approach to solicit a large amount of textual feedback from participants, and it allowed opportunities to discuss and clarify contributed notes with the breakout group members.

One Jamboard was used for each major section of a research report to ensure that the group would focus discussion on potential QRPs and IRPs that may occur in each major phase of the research process. These were (1) participants and setting; (2) dependent variables and measures; (3) research design; (4) independent variables; (5) analyzing and reporting results; (6) discussion/conclusion; (7) and introduction/abstract. The top half of each Jamboard contained a space for participants to post notes for potential QRPs in each area. The bottom half of each slide contained a space for participants to post notes for potential IRPs. Each of the microconference Jamboards are posted on the project’s OSF site: https://osf.io/mvy6c.

For each slide, the facilitator asked participants to post notes identifying ways that procedures and reporting practices related to the section of the manuscript may inflate effects, make results appear more generalizable than they actually are, and/or enhance socially validity beyond what is warranted (i.e., QRPs). This was followed by a discussion of the group’s responses. During the discussion, participants could revise their notes and contribute additional notes. Participants then were asked to post notes about potential IRPs that could reduce these kinds of invalid conclusions; again, initial writing of notes was followed by discussion, revision, and additions. If needed, each facilitator provided potential examples of QRPs and IRPs to assist participants with idea generation. A final Jamboard allowed participants to post notes about QRPs and IRPs that were not previously shared. Following the breakout group activities, the whole group reconvened for 35-min to solicit participant perspectives including their perceived value of the day’s experiences, their concerns, and guiding recommendations for advancing the project.

### Analysis of Microconference Data

A total of approximately 2,000 Jamboard notes identifying QRPs and IRPs were provided by the participants. We exported the notes to a Microsoft Excel file for each microconference group. Each file contained a list of participant comments that were organized according to sections of a manuscript or stages of research (e.g., participants and setting, analyzing and reporting results) and whether the comment was a suggested QRP or IRP. Given that participants were asked to post notes on potential QRPs and their corresponding IRPs for each section of a manuscript on the same Jamboard page, we were able to link QRPs with corresponding IRPs in our data analysis process.

We used an inductive or grounded theory approach (Linneberg & Korsgaard, 2019; Saldaña, 2021) to analyze the microconference data to generate our preliminary QRP-IRP list. This approach is characterized by direct coding of qualitative data to organize participant responses into categories that share thematic similarities. Thus, we followed an iterative analysis-synthesis process with the goal of identifying all unique QRPs and IRPs in the corpus of notes. Using Microsoft Excel and Atlas.ti qualitative analysis software program, the first four authors used open coding to group notes into thematically similar categories. Then, using axial coding, we generated a concise statement that captured the common theme of the set. For example, several participants noted that omission of participant data from a research report constituted a QRP, and thus these were categorized together as one QRP related to selective reporting of participants. We then paired each QRP with a corresponding IRP. When a QRP did not have a corresponding IRP or vice versa, we generated an IRP that conceivably addressed it to ensure all QRPs and IRPs had a corresponding practice. We then categorized clusters of QRP-IRP pairs into categories that aligned with broader components of conducting and reporting research (e.g., contextualizing and summarizing the study, experimental design).

In addition, through a selective coding process comprised of a series of discussions among the authors, we omitted participant notes that were incongruent with the conceptualization of QRPs and IRPs presented during the microconferences and detailed in this article. First, we excluded statements that represented desired or quality features of studies that were not relevant to addressing QRPs. For example, several notes identified a need to conduct more SCED research in practice settings with naturalistic implementers. Although an important area for development (see Carr & Horner, 2007), the choice of settings and implementers for any study is highly contextual and not necessarily reflective of the presence or absence of QRPs. Likewise, we omitted notes reflecting desirable research practices that were vague or general in nature, such as improving the quality of treatment fidelity measurement and quality of writing of the abstract and introduction. We also excluded notes representing potentially desired practices that did not have a clear link to a specific QRP, such as preregistering studies, as our aim was to identify IRPs linked to specific QRPs as alternative responses.

We did not include suggestions aimed at preventing QRPs or enhancing IRPs at the level of editorial procedures, research and publication guidelines, or research policies. For example, developing journal submission guidelines for completely describing participants might support adherence to IRPs, but is not one itself. We recognize, however, that such recommendations are important and will be a central part of the behavior change process to disseminate IRPs, and thus we noted them for later reference.

Practices that are controversial within the single-case research community, where there is a lack of consensus on whether they are beneficial, were also not considered QRP or IRPs. For example, the suggested QRP, “In multiple baseline designs, failing to randomly assign tiers to order of receiving intervention” was not considered a QRP because there is no consensus on random assignment of participants to tiers in a multiple baseline study (Ledford et al., 2018). Finally, scientific misconduct and explicit ethical violations were not considered QRPs and they are addressed in other ways, such as through institutional review boards or other research oversight entities. For example, data fabrication is research fraud and a form of scientific misconduct. However, the recognition of QRPs and IRPs may reduce researchers’ degrees of freedom and promote transparency leaving less room for conflicts of interest to influence research conclusions.

## Results

Our analysis resulted in 75 QRP-IRP pairs. We then reviewed and discussed the list with our advisory group, which resulted in further refinement of the items including splitting, combining, deleting, and rewording items for clarification/specificity. This reduced the list to 64 QRP-IRP pairs, which we organized in six broad categories (see Table 2). A brief description of each category with examples of QRP-IRP pairs follows.

**Table 2:** Questionable and Improved Research Practices in SCED Identified through the Microconference Process

**I. Contextualizing and Summarizing the Study in the Abstract, Introduction, and Discussion**	
**Questionable Research Practice**	**Improved Research Practice**
a. Overstating desired results and/or downplaying negative results in abstract	a. Accurately summarizing results in abstract: Neither overstating desired results nor downplaying undesired results
b. Failing to clearly state type of study (e.g., conceptual/systematic replication, part of dissertation, pre-registered, inductive/deductive) in the abstract	b. Clarifying the type of study being reported (e.g., conceptual/systematic replication, part of dissertation, pre-registered, inductive/deductive) in the abstract
c. Misrepresenting the literature in introduction or discussion, including inaccurate use of citations	c. Accurately representing previous literature in introduction and discussion. Accurately citing relevant studies
d. Using a label for the treatment that is not consistent with the actual operations or historical name for the treatment	d. Ensuring that treatment is labeled in a manner that clearly conveys actual operations and/or previously used terms
e. Overstating results: mismatch between data and statements about the data	e. Describing results in ways that are consistent with data patterns
f. Making statements in discussion section that are not fully supported by results including: i. overgeneralizing ii. overstating strength, clarity, and/or consistency of results; and iii. asserting high confidence in claims that are unjustified or otherwise unsupported by results, including implications for practice	f. Ensuring that all statements in discussion are fully supported by results
g. Failing to discuss and interpret negative and/or null results	g. Appropriately discussing and interpreting negative and/or null results
h. Failing to discuss how the current research relates to previous research	h. Clearly linking and discussing relations to previous research
i. Identifying only superficial limitations in discussion section	i. Discussing the most important limitations and drawing out their implications
j. Omitting known confounds in discussion of limitations	j. Reporting all known confounds and discussing them among limitations
**II. Participants, Settings, and Procedures**	
**Questionable Research Practice**	**Improved Research Practice**
a. Failing to clarify procedures that were determined a priori versus those adjusted during data collection	a. Clearly describing which procedures were determined a priori and which were adjusted during data collection
b. Failing to explicitly report all recruitment and selection factors, criteria, and procedures. This includes: i. factors that may be subtle, judgmental, or informal; ii. any convenience factors such as likelihood of attendance; iii. lack of nonrelevant behavior challenges, or language spoken; iv. how selections were made among qualified potential participants, incentives for participation; and v. systems-level selection (i.e., how the system in which you are working made some potential participants available or unavailable)	b. Explicitly reporting all recruitment and selection factors, criteria, and procedures. This includes: i. factors that may be subtle, judgmental, or informal; ii. any convenience factors such as likelihood of attendance; iii. lack of nonrelevant behavior challenges, or language spoken; iv. how selections were made among qualified potential participants, incentives for participation; and v. systems-level selection (i.e., how the system in which you are working made some potential participants available or unavailable)
c. Failing to report that modifications of recruitment and selection factors, criteria, and procedures were made during the study	c. Fully describing a priori recruitment and selection factors, criteria, and procedures, and any modifications that were made during the study
d. Selectively supporting (or failing to support) participants’ continued participation in the study based on their performance patterns	d. Providing all participants with equal support to continue participating in the study
e. Failing to report the number participants who began baseline sessions and each subsequent phase, and reason for attrition for those who leave the study prior to completion	e. Reporting number of participants beginning baseline and each subsequent phase of the study, and reasons for attrition. Reporting may include full data on all variables or an explanation of why this is not appropriate
f. Failing to fully report participant characteristics potentially affecting the DV or responsiveness to the IV, including: i. demographics (e.g., SES, race, ethnicity, language, disabilities, age); ii. other behaviors relevant to sensitivity to the IV and/or performance on DV, especially. those that may be cited in conclusions as an explanation of performance; co-morbidities (and how they were identified); and iii. pre-baseline performances that indicate need for the intervention (if any)	f. Fully reporting all participant characteristics potentially affecting the DV or responsiveness to the IV, including: i. demographics (e.g., SES, race, ethnicity, language, disabilities, age); ii. other behaviors relevant to sensitivity to the IV and/or performance on DV, especially. those that may be cited in conclusions as an explanation of performance; iii. co-morbidities (and how they were identified); and iv. pre-baseline performances that indicate need for the intervention (if any)
g. Failing to fully report participants’ previous experience with the researchers and/or related interventions, including in studies or service delivery	g. Fully reporting participants’ previous experience with the researchers and/or related interventions, including in studies or service delivery
h. Failing to fully report participant characteristics of any kind that make participants appear to be more homogenous, more in need of treatment, more typical of a given population, etc	h. Fully reporting participant characteristics of any kind relevant to homogeneity, need for treatment, typicality of a given population, etc
i. Failing to fully describe relevant (i.e., functional) features of the setting(s) including: i. the degree to which a setting is typical of the terms used to name it (e.g., school, classroom); ii. programming in place that provides background to the intervention (e.g., tier 1 PBIS, rate of reinforcement); iii. nonparticipants in the room; iv. resources and supports; v. changes in the setting that occur during the study; and vi. the researcher’s previous relationship with setting	i. Fully describing relevant (i.e., functional) features of the setting(s) including: i. the degree to which a setting is typical of the terms used to name it (e.g., school, classroom); ii. programming in place that provides background to the intervention (e.g., tier 1 PBIS, rate of reinforcement); iii. nonparticipants in the room iv. resources and supports; v. changes in the setting that occur during the study; and vi. the researcher’s previous relationship with setting
j. Describing settings in ways that make them appear more typical or generalizable than they are	j. Describing settings in ways that accurately reflect how typical or generalizable they are
k. Failing to report training/experience of implementers of the intervention	k. Reporting training/experience of implementers of the intervention
l. Failing to report all materials relevant to implementation of the intervention and failing to carefully analyze what aspects of materials are relevant	l. Reporting all materials relevant to implementation of the intervention and carefully analyzing what aspects of materials are relevant
m. Omitting details about interventionist training (e.g., coaching, booster sessions, feedback)	m. Reporting all relevant interventionist credentials and training (e.g., coaching, booster sessions, feedback)
n. Failing to disclose researchers’ involvement in sessions (as distinct from other implementers)	n. Clearly describing researchers’ involvement in sessions (as distinct from other implementers)
**III. Dependent Measures, Interobserver Agreement, and Treatment Fidelity**	
**Questionable Research Practice**	**Improved Research Practice**
a. Failing to clearly report any changes in operational definitions, data collection, and/or measurement procedures that were made during the study and analysis	a. Clearly reporting any changes in operational definitions, data collection, and/or measurement procedures that were made during the study and analysis
b. Failing to report all data on all measures or provide a specific rationale for not reporting. This includes failing to report: i. all dependent measures that were administered; ii. all generalization and maintenance measures that were administered; iii. all social validity measures that were administered; iv. all treatment fidelity measures that were administered; v. all data points collected in all phases and conditions (unless specific acknowledgement and explanation is given for dropping data points)	b. Reporting all data on all measures or provide a specific rationale for not reporting. This includes reporting: i. all dependent measures that were administered; ii. all generalization and maintenance measures that were administered; iii. all social validity measures that were administered; iv. all treatment fidelity measures that were administered; v. all data points collected in all phases and conditions (unless specific acknowledgement and explanation is given for dropping data points)
c. Selecting less conservative/accurate IOA calculation methods more likely to yield high agreement	c. Computing IOA with the most fine-grained comparison that are relevant to the measure
d. Failing to collect and report disaggregated IOA data for each participant, in each phase, on each measure	d. Reporting disaggregated IOA data for each participant, on each measure, in each phase
e. Using nonindependent IOA data collectors (i.e., one data collector influences the other)	e. Ensuring that IOA data collectors cannot influence one another’s scores
f. Collecting IOA data until acceptable agreement is obtained, and then stopping	f. Establishing IOA data collection schedule a priori and report any adjustments to the schedule
g. Not using blinded data collectors when it is possible do so	g. Ensuring that data collectors are blind to condition when it is possible to do so and reporting procedures for this
h. Collecting procedural fidelity data by biased observer or via self-observation	h. Collecting procedural fidelity data by an unbiased, independent observer
i. Reporting aggregated treatment fidelity data that does not represent which features were (not) consistently implemented	i. Reporting treatment fidelity results at a sufficiently disaggregated level for reader to understand which features were more/less consistently implemented
j. Failing to collect treatment fidelity data that is representative of each phase/condition and report it disaggregated by phase/condition	j. Collecting treatment fidelity data that is representative of each phase/condition and report it disaggregated by phase/condition
**IV. Experimental Design**	
**Questionable Research Practice**	**Improved Research Practice**
a. Failing to report procedures for all sessions, including instances of interaction with researchers or research procedures, whether or not data were collected. This includes “warm up sessions”	a. Reporting procedures for all sessions, including instances of interaction with researchers or research procedures, whether or not data were collected. This includes “warm up sessions”
b. Selectively reporting phases/conditions: Failing to describe procedures and report data from all phases and conditions that were conducted	b. Fully reporting all phases/conditions that were implemented during the study
c. Using inconsistent criteria across conditions/phases for determining whether a session will be conducted on a given day	c. Using consistent criteria across conditions/phases for determining whether a session will be conducted on a given day
d. Creating baseline conditions that do not approximate pre-baseline/naturalistic levels of behavior, resulting in magnified apparent changes in behavior in subsequent conditions, and this is not clearly reported	d. Using baseline conditions that approximate pre-baseline/naturalistic levels of behavior or, if not, this is clearly reported
e. Using design features to inflate results from statistical analysis (e.g., extending a phase to push the phase mean in desired direction)	e. Refraining from using design features to inflate results from statistical analysis (e.g., extending a phase to push the phase mean in desired direction)
f. Failing to provide a thorough description of the IV including: i. all relevant aspects of the IV (e.g. all components of a multicomponent package) ii. modifications made during the study; and iii. modifications for individuals or modifications to treatments used in previous studies	f. Providing a thorough description of the IV including: i. all relevant aspects of the IV (e.g. all components of a multicomponent package); ii. modifications made during the study; and iii. modifications for individuals or modifications to treatments used in previous studies
g. Failing to describe known changes that coincide with the IV but are not considered to be part of it (i.e., confounds; e.g., changes in schedule, staffing, locations)	g. Describing known changes that coincide with IV but are not considered to be part of it (i.e., confounds; e.g., changes in schedule, staffing, locations)
h. Failing to state whether phase change criteria were pre-specified	h. Reporting prespecified phase change criteria or lack thereof
i. Failing to fully describe the criteria used to determine timing of phase changes	i. Fully describing criteria used to determine timing of phase changes
j. Assigning of tiers/participants to a multiple baseline design based on anticipated responding/sensitivity/stability	j. Assigning order of phase changes to tiers in a multiple baseline design based on prespecified and clearly reported criteria
k. Failing to report how decisions were made about participant assignment to tiers in a multiple baseline	k. Reporting how decisions were made about participant assignment to tiers in a multiple baseline
l. Failing to clearly report whether multiple baseline designs are concurrent or nonconcurrent and specifics on how sessions were timed or synchronized across tiers	l. Clearly reporting in multiple baseline designs the temporal relation among tiers including concurrence and how closely the sessions are synchronized across tiers
m. Failing to report details about baseline conditions that clearly demonstrate presence or absence of relevant variables that may be influencing responding	m. Reporting details about baseline conditions that clearly demonstrate presence or absence of relevant variables that may be influencing responding
n. Failing to report passage of real time between experimental sessions	n. Depicting passage of real time on graph or report in narrative explanation of real time
o. Failing to clearly indicate multiple sessions per day in graphed data or narrative text	o. Clearly indicating in graph or text which sessions occurred on a single day
p. Failing to clearly indicate temporal gaps between sessions that could influence interpretation	p. Clearly indicating (in graph or text) temporal gaps between sessions that could influence interpretation
q. Reporting that a randomized component of design was randomized when process was not truly random (e.g.,“do overs”)	q. Transparently and rigorously reporting randomization procedures
**V. Data Analysis and Reporting**	
**Questionable Research Practice**	**Improved Research Practice**
a. Selectively graphing/reporting data (e.g., omitting outliers; disguising missing data) without explicit justification	a. Explicitly reporting how missing data and outliers are dealt with and discussing potential implications for interpretation
b. Graphing data in ways that misrepresent results such as manipulating scale, axes, design, or other presentations that improve the appearance of a functional relation	b. Graphing data in ways that accurately represent results
c. Selectively reporting statistical analyses that show better results	c. Reporting all analyses conducted (preliminary analyses may be on online supplement)
d. Failing to provide rationale for selection of statistics	d. Providing rationale for selection of statistics
e. Failing to explicitly describe visual analysis procedures	e. Explicitly describing visual analysis procedures
f. Failing to explicitly state what data features and comparisons contribute to conclusions about the data	f. Explicitly stating what data features and comparisons contribute to conclusions about the data
g. Failing to prespecify data analysis plan/protocol and report any subsequent changes to the plan	g. Prespecifying the data analysis plan/protocol, and report changes to the analytic plan
h. Selectively displaying results (i.e., displaying data that show favored results more prominently; e.g., in graphs) and data that suggest less favored results less prominently (e.g., in tables or text)	h. Displaying results in graphs, tables, and text based on importance to research questions and clarity
i. Selectively describing data features that appear to support favored conclusions	i. Describing features of data objectively (without bias regarding favored results)
j. Aggregating/disaggregating data to enhance apparent results without full reporting of the process	j. Reporting when aggregating or disaggregating data that may affect interpretation of results
k. Relying on statistical analysis to make causal inferences	k. Using visual analysis rather than statistical analysis for making causal inferences
l. Failing to make raw data available in supplemental materials (including time elapsed between sessions and demographic information when possible)	l. Reporting raw data in supplementary materials (including time elapsed between sessions and demographic information when possible)
**VI. Study Level**	
**Questionable Research Practice**	**Improved Research Practice**
a. Failing to submit methodologically strong studies that find null results	a. Submitting methodologically strong studies that find null results

*DV* dependent variable, *IV* independent variable

### Contextualizing and Summarizing the Study in the Abstract, Introduction, and Discussion

These 10 items focused on how the study is framed within the introduction and abstract of the research report, and how the outcomes of the study are interpreted in the discussion. The statements were specific to each of these sections. For example, the QRP in one pair was, “overstating desired results and/or downplaying negative results in abstract” and, the corresponding IRP was, “accurately summarizing results in abstract: neither overstating desired results nor downplaying undesired results.” Another QRP was, “failing to discuss and interpret negative and/or null results” and the corresponding IRP was, “appropriately discussing and interpreting negative and/or null results.”

### Participants, Settings, and Procedures

The next 14 items addressed how researchers report information about participants, settings, and procedures that potentially influence results of an experiment. This included recruitment and selection of participants; participant, setting, and implementer characteristics; and modifications to an intervention during a study. One pair related to participant recruitment included the QRP, “Failing to report that modifications of recruitment and selection factors, criteria, and procedures were made during the study” with the IRP, “Fully describing a priori recruitment and selection factors, criteria, and procedures, and any modifications that were made during the study.” A different example related to participant characteristics was, “Failing to fully report participant characteristics potentially affecting the dependent variable or responsiveness to the independent variable” and was contrasted with, “Fully reporting all participant characteristics potentially affecting the dependent variable or responsiveness to the independent variable.”

### Dependent Measures, Interobserver Agreement, and Treatment Fidelity

These 10 items focused on researcher choices regarding dependent variables and measurement systems, and procedures for conducting and reporting interobserver agreement and treatment fidelity. With respect to dependent measures, one QRP was, “Failing to report all data on all measures or provide a specific rationale for not reporting” with the corresponding IRP, “Reporting all data on all measures or provide a specific rationale for not reporting.” In terms of interobserver agreement, one QRP was, “Using non-independent IOA data collectors (i.e., one data collector influences the other)” and the corresponding IRP was, “Ensuring that IOA data collectors cannot influence one another’s scores.” With regard to treatment fidelity, one QRP-IRP pair was, “Collecting procedural fidelity data by biased observer or via self-observation.” and, conversely, “Ensuring that data collectors are blind to condition when it is possible to do so and reporting procedures for this.”

### Experimental Design

These 17 items pertained to procedures and reporting of SCED experimental designs. These included baseline procedures, independent variables, experimental conditions/phases, temporal aspects of experiments, and other factors that could influence study outcomes. For example, one QRP-IRP pair was “Creating baseline conditions that do not approximate pre-baseline/naturalistic levels of behavior, resulting in magnified apparent changes in behavior in subsequent conditions, and this is not clearly reported” and the alternative, “Using baseline conditions that approximate pre-baseline/naturalistic levels of behavior or, if not, this is clearly reported.” A different QRP-IRP pair included, “Failing to clearly indicate multiple sessions per day in graphed data and text” and was contrasted with, “Clearly indicating in graph or text which sessions occurred on a single day.”

### Data Analysis and Reporting

A set of 12 items related to procedures employed by researchers to analyze and report data from an experiment. Items pertained to reporting missing data, reporting data analysis plans, depicting data in graphs, and statistical analysis, among others. For instance, one QRP regarding missing data was, “Selectively graphing/reporting data (e.g., omitting outliers; disguising missing data) without explicit justification” and the alternative IRP was, “Explicitly reporting how missing data and outliers are dealt with and discussing potential implications for interpretation.” With respect to visual analysis of graphs, one QRP-IRP pair was, “Failing to explicitly describe visual analysis procedures” and conversely, “Explicitly describing visual analysis procedures.” Finally, with regard to statistics, a QRP-IRP pair included, “Failing to provide rationale for selection of statistics” and alternately, “Providing rationale for selection of statistics.”

### Study Level

One item did not readily fit into the other five areas. This item pertained to study level results reporting. In particular, the QRP was, “Failing to submit methodologically strong studies that find null results” and the IRP was, “Submitting methodologically strong studies that find null results.”

## Discussion

Researchers who employ group experimental designs have identified QRPs that compromise the validity of scientific research (Anvari & Lakens, 2018; John et al., 2012; Makel et al., 2021; Pashler & Wagenmakers, 2012; Simmons et al., 2011). However, the SCED research community has not yet identified a comprehensive list of QRPs that are specifically applicable to its methodology, rendering unclear what constitutes questionable practice, improved practice, and how the latter might be promoted. The purpose of this project was to systematically identify QRPs and corresponding IRPs for SCED research by soliciting perspectives of leading SCED researchers through a microconference process followed by qualitative analysis. We found participants had numerous and, often, similar concerns about researcher practices they considered questionable. Likewise, they expressed shared perspectives about what constitutes improved practices that might be leveraged to mitigate QRPs.

It should be noted that the list of QRPs with corresponding IRPs was much longer and broader than those often found in group comparisons research. For example, Makel et al. (2021) identified 10 QRPs in education research relying on group experimental designs, whereas we identified 64 QRPs and corresponding IRPs in SCED. One potential reason for this difference relates to the process used for identifying and describing QRPs in prior work versus our project. In particular, QRPs described in previous articles were often identified by the authors and not based on any systematic process (Banks et al., 2016; Gerrits et al., 2019; John et al., 2012; Makel et al., 2021; Matthes et al., 2015; Nosek et al., 2012; O’Boyle et al., 2017). In contrast, the QRPs identified in this article were based on discussions among an experienced group of research experts with the goal of generating an exhaustive list. Their diverse perspectives likely resulted in a more comprehensive and therefore larger list than we could have generated without their input.

In addition, investigations of QRPs in group research have tended to focus solely on identifying potentially problematic research practices associated with data analysis and reporting (e.g., post-hoc data manipulation, selective reporting; John et al., 2012). In contrast, we intentionally solicited discussion of QRPs and IRPs related to all sections of a research report from the abstract and introduction through conclusions, encompassing QRPs that may occur before, during, and after a study. In response, participants contributed numerous QRPs and IRPs associated with phases of the research process beyond data analysis. For example, our analysis yielded 10 pairs related to the abstract, introduction, and discussion, areas not considered in much of the previous work on QRPs (for an exception, see Gerrits et al., 2019).

Differences between group comparison and SCED methodology may also account for our more extensive list of QRPs. For example, SCED allows researchers to modify their independent variables during the study when participants appear unresponsive, and to continue modification until a treatment effect is apparent (Mechling et al., 2018). Likewise, SCED allows for response-guided decisions about when to introduce and/or withdraw an intervention to demonstrate, confirm, and replicate a treatment effect (Barton et al., 2018). Overall, these features of SCED may result in increased degrees of freedom that have many important benefits for research, but also may be subject to additional QRPs.

Our working hypothesis at the outset was that quality research indicators and open science practices are complementary to, but not entirely sufficient for preventing QRPs and promoting IRPs in SCED research. Our findings suggest there indeed appears some degree of overlap between published quality indicators (e.g., Cook et al., 2015; Horner et al., 2005; Kratochwill et al., 2023), open sciences practices (e.g., Cook et al., 2018; Nosek et al., 2012; Tincani et al., 2024), and the IRPs presented in Table 2. For example, Horner et al. (2005) emphasized reporting details of the study design with replicable precision (e.g., describing participants and the process for selecting them; using a design that yields results indicative of experimental control), which correspond with the IRPs described in sections II, III, and IV of Table 2. Formal alignment or integration of IRPs with quality indicators may clarify how they complement each other and may suggest further refinement of future quality indicators.

Likewise, open science practices appear to align with at least some of the IRPs we identified. For example, open data practices may reveal selective reporting of participants and/or dependent variables (i.e., open data practices may reveal that a researcher omitted from their published report some or all results for a particular participant, condition, or dependent variable). However, the IRP describing an alternative to selective reporting is more specific and requires researchers to explicitly report the number of participants who began a baseline condition along with reasons for removal or attrition. Likewise, open protocols associated with open science practices may clarify what the intervention procedures were and whether fidelity data were bolstered with superfluous steps. However, inflating fidelity metrics can be prevented via an IRP that requires only relevant procedures be included in the protocol. Open science practices and IRPs highlight how SCED researchers can clarify which methodological decisions were established prior to the study and which occurred during the course data collection and analysis. For example, study preregistration allows opportunities to document how a SCED study was initially planned and in what specific ways adjustments were made during and after the study, including those related to data analysis and reporting. Further work may elaborate how specific open science practices can act as IRPs and improve the validity of SCED in specific ways.

### Limitations

There are several limitations to this project. Our synthesis process was deliberate and iterative, consistent with qualitative research, which led to refined criteria to distinguish QRPs from other issues and controversies in SCED. For example, several participant comments focused on the review and editorial process, limited diversity of participants in the SCED literature, ideal experimental practices (e.g., number of data points in a condition; intervention agents natural to the study setting), and methodological controversies (e.g., blinding data collectors, randomized assignment to tiers in multiple baseline design studies). Although we believe it valuable to distinguish these practices from QRPs and IRPs as we conceptualized them, we recognize SCED researchers may not universally agree on these distinctions. In addition, our process included the development of a small subset of IRPs for questionable practices for which no improvement suggestions were explicitly provided by participants. Although we developed these additional IRPs based on the inverse behavior of the QRP, such IRPs reflect our inference of specific practices not offered by participants. Nonetheless, we believe our iterative process in conjunction with guidance from our advisory board members demonstrates sufficient credibility of our findings and represent specific practices consistent with the general concept of IRPs.

Related, we sought to describe each QRP-IRP pair in Table 2 as concisely as possible; however, many QRP-IRP pairs lack sufficient elaboration, including examples, to be useful as an implementation guide for SCED researchers. For instance, implementation of the IRP, “Describing results in ways that are consistent with data patterns” is unique according to the research questions posed, the type of designed used, the nature of the data collected, and other study specific variables. Therefore, we concede the need for future work aimed at providing expanded guidance to researchers for how best to implement IRPs based on the unique circumstances of their research projects.

An additional and arguably significant limitation of our study relates to whether our findings represent the general consensus of SCED researchers. Although we recruited broadly such that our participants represented different disciplines, career stages, roles, and demographic characteristics, many prospective participants were not available to contribute, thus our sample was not a diverse as would be ideal. However, nearly 60% of the 115 invited participants accepted our invitation to participate and contributed approximately 2,000 notes for our analysis and synthesis. This high degree of participation and contribution resulted in saturation of data, supporting the credibility of our findings. That is, through our coding process we were able to identify distinct QRP-IRP pairs aligned to broader categories (e.g., experimental design, dependent variables) to the point at which no new information could be gleaned from our dataset (Saldaña, 2021). Still, our method does not inform conclusions about consensus about QRP-IRP pairs among the SCED community. Related to this, our method did not allow interpretations about the relative severity or frequency of each QRP, nor any inferences about the potential (in)feasibility of each IRP. The extent to which QRPs affect SCED and the potential feasibility of the identified IRPs therefore remains unclear.

### Future Directions for the Field

Results obtained from this project serve as an initial step toward better understanding QRPs and IRPs in SCED research, but several questions remain to be addressed in the future. First and perhaps most important, future research should investigate the extent to which consensus exists for each QRP-IRP pair. Our results do not reflect how often any specific QRP or IRP was offered by participants, nor do they indicate whether most SCED researchers agree that any of the identified practices represent questionable or improved scientific practices. A survey study with a large number of participants from various disciplines that often publish SCED research might reveal whether consensus exists for each QRP-IRP, and reveal whether (or in what contexts) a proposed QRP actually is not viewed as such by some or most of the SCED research community. Such a study could also ask participants to rate their perceptions about the severity of each QRP (i.e., the potential negative effect of each QRP on the advancement of scientific knowledge). Related to this, a survey also could ask participants about whether the proposed IRPs are both feasible and sufficient to address its corresponding QRP. We view this as a critical process prior to suggesting that the list of QRPs and IRPs would be sufficiently validated to warrant dissemination. Therefore, it would be premature to develop elaborate examples and other resources that would support researchers in adopting IRPs. Recommendations may be more readily accepted and enacted following evidence demonstrating which QRPs and IRPs are supported by a consensus of SCED researchers.

Future investigators also might consider how modifications to the peer-review and editorial decision-making processes might be adjusted to encourage IRPs as replacement behavior for QRPs. For example, consistent with Shadish et al. (2016), participants in our project suggested that datasets with equivocal or otherwise unclear results are sometimes omitted from reports of SCED research (i.e., selective reporting). If SCED researchers largely agree that selective reporting is a QRP, then researchers might investigate ways to change the review and editorial processes to promote more complete and transparent reporting. In particular, researchers might measure attitudes and beliefs about the value of equivocal results and the potential knowledge gained by reporting equivocal findings and the harms associated with omission. Also, a series of survey studies could examine how often SCED researchers are instructed to selectively report during the editorial process as a way of monitoring changes in reviewer and editor recommendations over time. Such research could mitigate the file drawer effect (i.e., submission of noneffect studies) and stimulate changes in journal policies in ways that reduce the potential impact of QRPs on the validity of the SCED research corpus.

Related to this, if publications function as reinforcement for QRPs, then it seems reasonable to focus on training peer reviewers to recognize QRPs and how to guide authors toward transparent reporting—changing the contingencies and providing training to contact the new reinforcer. Thus, researchers might investigate whether training for peer reviewers improves their recognition and mitigation of QRPs in SCED research. Related to this, the SCED research community should consider whether and what changes in journal editorial policies might mitigate QRPs while promoting IRPs. Similar past efforts to influence reviewer and editorial policy have led to shifts in SCED research norms and contributed valuable knowledge in behavior analysis (e.g., social validity; Schwartz & Baer, 1991).

A final potential avenue for future research might focus on formal alignment of IRPs with methodological quality indicators and open science practices. As discussed above, quality indicators and open science practices complement but may not entirely overlap with IRPs. Formal alignment may reveal where specific foci overlap and are distinct, which may simplify how SCED researchers might more readily advance the methodology. For example, if specific quality indicators and open science practices preclude specific QRPs (e.g., detailed reporting and rationale for decisions, open data, open research protocols), then the list of IRPs may be shortened and adoption more feasible. On the other hand, areas that do not overlap may help clarify which QRP-IRPs command explicit attention from researchers, reviewers, and editors to maintain integrity of the SCED corpus. In addition, alignment between these different frameworks may support advances toward clarifying a conceptual framework for QRPs in SCED research.

## Conclusion

Advances in scientific knowledge often depend on advances in scientific methodology and reporting. The research described here is a contribution to the ongoing process of refinement of SCED in service of improved validity of conclusions and practical impact. It borrows from group comparison literature the concept of QRP and the understanding that researchers are influenced by complex contingencies of reinforcement that may not perfectly align with validity. Recognition of QRPs is an additional step in the historical elaboration of research methods. We have developed the concept of IRP to provide clear alternative behaviors that can be promoted as replacements for QRPs. We also developed a novel method for identifying QRPs and IRPs based on discussion among a broad group of researchers. These participants considered each section of a research report, not limiting the inquiry to data analysis issues, another departure from previous work. We found that SCED researchers offered numerous QRPs and IRPs associated with each section of a manuscript and that there was a great deal of consensus among contributors. This set of QRPs and IRPs is not encompassed by or redundant with either open science practices or existing quality indicators, but rather they appear to complement these other kinds of methodological guidelines. This study represents an initial step in identifying and addressing QRPs and IRPs for SCED; however, the results reported here should be considered preliminary and tentative until they are further validated.

## Data Availability

The study's data are available at https://osf.io/mvy6c/.
